# Diagnosis of Neuralgia and Neuritis

**Published:** 1847-03

**Authors:** 


*Diagnosis of Neuralgia and Neuritis.*—Although in some cases the symptoms of these two affections of the nerve are so nearly similar, that it is difficult to distinguish at first sight the one from the other, the confusion will cease in general, if, instead of inquiring into the actual condition of the patient, our inquiries are directed to the prior history of the attack, its progress, and exciting cause. While, in fact, *neuralgia* is a very common affection arising without appreciable cause, or from causes the more opposite in character, *neuritis* is a rare affection, and is determined by causes which are readily appreciated. In analyzing the best authenticated cases of neuritis, it will be found that, with the exception of some few cases, in which it followed parturition, neuritis has almost constantly been produced by physical lesions of the nerve—such as wounds, punctures, contusions, ligature, compression by a tumor, &c.; in fact, neuritis is always, or nearly always, the result of mechanical injury, while neuralgia orginates spontaneously, and depends upon a particular, and little understood, condition of the economy. But if it is sometimes possible and useful to establish this distinction in practice, especially in neuralgia and neuritis of recent date, it cannot be denied, that in a certain number of cases of chronic neuritis, the distinction becomes impossible; for although it has been ascertained that neuralgia of very old standing (thirty or forty years for example,) may have preserved its original character throughout, and yet left no traces of disease after death, it happens in the majority of cases, that under the influence of the repetition of the paroxysms, the texture of the nerve eventually becomes altered to such a degree as to render it quite impossible to decide whether the inflammation has been secondary, or has depended upon an original neuritis. These cases show the inutility of attempting a diagnosis in the chronic forms of the affections.—*Gazette Medicale de Paris,* No. 40, 1846.

